# Chitosan Microgels and Nanoparticles via Electrofluidodynamic Techniques for Biomedical Applications

**DOI:** 10.3390/gels2010002

**Published:** 2016-01-12

**Authors:** Vincenzo Guarino, Rosaria Altobelli, Luigi Ambrosio

**Affiliations:** Institute for Polymers, Composites and Biomaterials, Department of Chemical Sciences & Materials Technology, National Research Council of Italy, Mostra D’Oltremare, Pad.20, V.le Kennedy 54, Naples 80125, Italy; rosaria.altobelli@gmail.com (R.A.); ambrosio@unina.it (L.A.)

**Keywords:** chitosan, electrospraying, electrohydrodynamic atomization, drug delivery

## Abstract

Electrofluidodynamics techniques (EFDTs) are emerging methodologies based on liquid atomization induced by electrical forces to obtain a fine suspension of particles from hundreds of micrometers down to nanometer size. As a function of the characteristic size, these particles are interesting for a wide variety of applications, due to the high scalability of chemical and physical properties in comparison to the bulk form. Here, we propose the optimization of EFDT techniques to design chitosan systems in the form of microgels or nanoparticles for several biomedical applications. Different microscopy techniques (Optical, SEM, TEM) have been used to investigate the morphology of chitosan systems at multiple size scale. The proposed study confirms the high versatility and feasibility of EFDTs for creating micro and nano-sized carriers for cells and drug species.

## 1. Introduction

In the last decade, hydrogels in the form of capsules or particles have been largely used to deliver active molecules or living cells for therapeutic and cell-based disease treatments [1,2]. Their water affinity is generally attributed to the presence of hydrophilic groups—such as ether, amino, hydroxyl, sulfate and carboxyl—properly distributed along the polymer chains which contribute to the development of specific drug release profiles as a function of their macroscopic networks or confined state [3]. This peculiar capability, to generate a highly hydrated microenvironment, also allows for protecting sensitive drugs, thus preserving molecular stability prior to the delivery at the site of injury [4]. Moreover, this assures an efficient transport of biological substances, such as nutrients and products from cell metabolism, in and out of the hydrogels [5], which are fundamental to protect and sustain cell viability during the regeneration processes [6].

In this context, hydrogels have been recently engineered in the form of “microgels” to encapsulate stem cells in order to address their fate by controlling the diffusion of various molecular signals exerted by niche cells or the surrounding extracellular matter [7]. Moreover, they have been also processed in the form of nanoparticles and used as innovative drug delivery systems, owing to their unique properties to confine their main features (e.g., swelling, controlled molecular release) into a sub-micrometric units [8]. Recently, a large variety of synthetic hydrogels have been prepared with tailored and highly reproducible chemistry and physical properties, thereby providing the required degradation properties [9]. By a sage combination of different monomers or the incorporation of bio-functional units, it is possible to properly adjust polymer chain length and density in order to design hydrogels with customized functionalities in terms of degradation rate, swelling ratios, mechanical and transport properties [3,10]. However, natural hydrogels usually display a wide heterogeneity of chemical properties, compared to synthetic ones, strictly due to their natural origin, which limits the reproducibility and functionality of the materials, thus resulting preferably in terms of biological recognition. For this purpose, several works have demonstrated the excellent compatibility of naturally derived hydrogels such as polysaccharides in the presence of natural tissues [11]. Their good control of solute permeability and their ability to be easily injected directly into the defect size under non-toxic , allows preventing any local alteration of functionality of biomolecules, preserving viability of encapsulated cells [12]. The choice of a specific hydrogel is strictly dependent upon the administration strategy, molecular chemistry, and release profiles, which may address a more appropriate release profile driven by diffusion and/or degradation mechanisms. Recently, chitosan (CHI), used alone or in combination with synthetic polymers, has gained great attention due to its unique physicochemical properties, such as pH sensitivity, biocompatibility, low toxicity [13] and its degradability by human lysozyme [14], which make it an ideal material for cell and drug delivery systems.

Therefore, size and surface-to-volume ratio of particles—not only materials properties—drastically influence encapsulation/delivery mechanisms as a function of the specific processing route. In recent years, many synthetic and natural hydrogels have been fabricated in the form of micro and sub-micrometric carriers by using different technologies, *i.e*., emulsion, desolvation, ultrasound vibration, spray drying, air jet and electrospray. Among them, the Electro Spray (ES) technique—including Electro Hydro Dynamic Atomization (EHDA) and Electro Dynamic Spraying (EDS)—currently represents one of the most efficient methods to design cell and molecular carriers in the field of biomedical micro and nanotechnology [15]. This technique is based on the production of full or hollow spheres from a polymer solution, by applying a high voltage electric field. The principle of the electrospray is based on the ability of electric forces to charge solution droplets by deforming their interface until breaking them into smaller droplets in the micrometric/sub-micrometric range. The jet deforms and disrupts into droplets due mainly to electrical forces by the competition between coulomb forces related to surface charge and cohesive forces inside the droplet, without the administration of additional mechanical energy to reach the liquid atomization [16]. Charge and droplet size can be finely controlled to some extent by the applied voltage, polymer solution concentration, nozzle-collector gap, flow rate and needle diameter [17].

**Figure 1:**
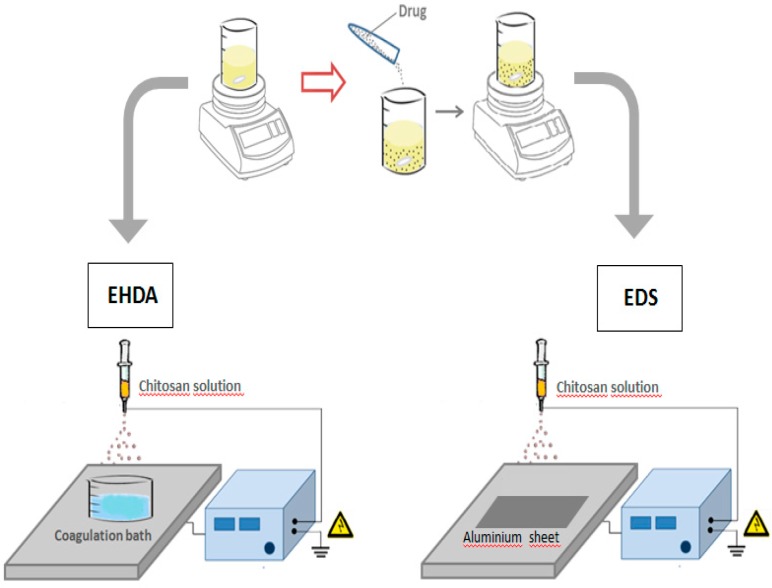
Schematization of EHDA and EDS processes for the fabrication of chitosan based microgels and nanoparticles.

According to the specific mode to collect polymeric droplets, two variants of the ES technique can be considered, EDS and EHDA respectively, as a function of the collector used (Figure 1):

(a) EDS involves the deposition of charged droplets on a grounded plate, by the breaking of polymer jet into nano-droplets under the solution overcharging conditions to form individual nanoparticles or agglomerates as a function of the local surface charge.
(b) EHDA is based on the deposition of charged droplets in a crosslinking agent solution—*i.e.*, calcium chloride (CaCl_2_) for alginate particles—prior to the solution overcharging, by the perturbation and cutting of polymer jet until the formation of microsized particles.

In this work, we investigate the potential use of ES technologies to manipulate CHI droplets at different size scale in order to design innovative micro or sub-microcarriers for cells and/or macromolecules to be used in the biomedical field. Particle morphology was preliminary investigated by optical microscopy at micrometric size scale, and by scanning (SEM) and transmission (TEM) electron microscopy at sub-micrometric size scale. Drug release profiles from different sized chitosan particles were evaluated at different pH via spectrometric analysis.

## 2. Results

### 2.1. Microgels

**Figure 2:**
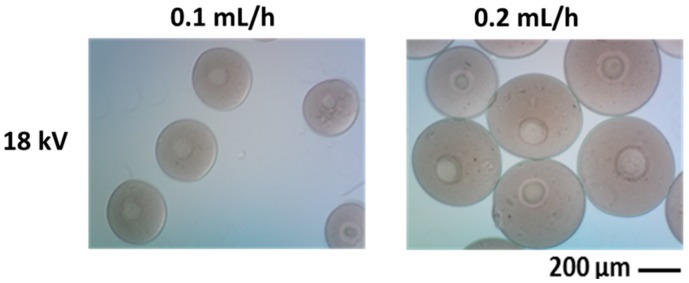
Chitosan microgels fabricated via EHDA: size variation via optical images as a function of flow rate.

Figure 2 shows the morphology of chitosan microgels obtained by using two different flow rates, 0.1 and 0.2 mL/h respectively. The optimization of process conditions allows producing narrowly dispersed gel-like units with sizes ranging from 169 to 253 μm. Independently upon the process parameters used, produced particles present a rounded shape which is imparted them once droplets are collected in the crosslinking bath. By controlling the flow rate, it is possible to modify particle size up to twofold increase, while further slight variation may be reached by tuning the applied voltage. In particular, it is possible to recognize a voltage threshold value corresponding to the starting condition to break polymer flow into smaller droplets. This value is strongly influenced by process parameters (*i.e*., flow rate) and materials properties (*i.e*., molecular weight, polymer concentration). In particular, increasing their variation may generate voltage shifts to higher values, thus negatively influencing particle size distribution.

### 2.2. Nanoparticles

**Figure 3:**
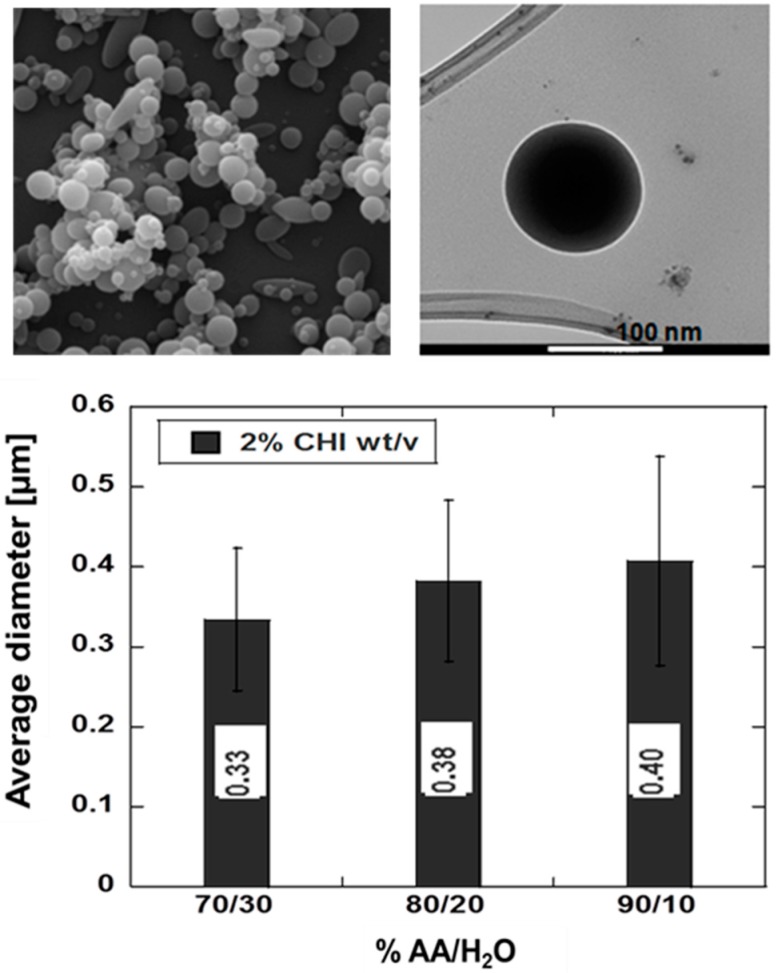
Chitosan nanoparticles fabricated via EDS: morphological analyses by SEM and TEM, and particle size measurement via image analysis as a function of acetic acid/water (AA/H_2_O) ratio.

Figure 3 shows chitosan nanoparticles fabricated by EDS technique. The process—simply schematized in Figure 1—allows producing monodisperse droplets by an appropriate definition of polymer solutions in terms of solvent/co-solvent ratios. SEM images clearly show sub-micrometric particles with a rounded shape and smooth surface. Accordingly, TEM shows isolated nanoparticles with aspect ratio—namely minor axis/major axis—close to one.

We verify that acetic acid/water ratio (*i.e*., 70/30 *v*/*v*, 80/20 *v*/*v* , 90/10 *v*/*v*) mainly influences the particle size. Image analysis on selected SEM images indicates a remarkable reduction of average diameters moving from 90/10 to 70/30 *v*/*v*, ranging from (0.41 ± 0.09) μm to (0.33 ± 0.13) μm. This phenomenon is ascribable to a decrease of the solution conductivity and the consequent decrease of the inter-ionic forces, according to the increment of the acetic acid concentration from 70% to 90%. In this case, particles show a well defined round-like shape with low polydispersivity in size but higher tendency to cluster formation. Clustering phenomena, mainly observed for 70/30 solvent/cosolvent ratios, are probably due to the slower evaporation of the acetic acid/water mixture during the process and to the greater surface area/volume ratio exhibited by smaller particles.

As the flow rate increases from 0.1 to 0.3 mL/h, the particle size coherently increases, from (0.25 ± 0.03) μm to (0.31 ± 0.11) μm. It is observed that, at higher flow rates, coalescence phenomena and the formation of aggregates are prevalent. Solvent tends to not sufficiently evaporate, so that nanoparticles tend to aggregate onto the collector, thus splashing onto the particles layeralready deposited. This effect may be neglected at lower flow rates due to a more efficient evaporation of solvents. Particle size is also influenced by the applied voltage. For higher voltage values (e.g., 25–28 kV), the jet become sunstainable, not allowing the control of particles size, thus promoting the formation of clusters and irregular shapes of particles.

**Figure 4:**
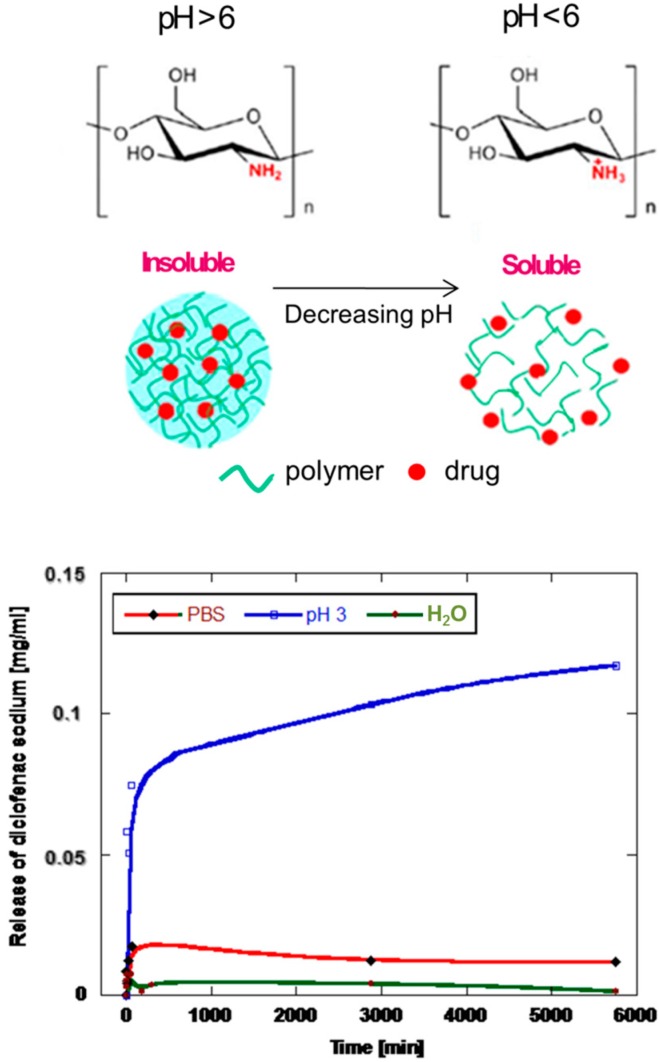
Release of sodium diclofenac from chitosan nanoparticles at different pH conditions.

The *in vitro* release of drugs from CHI nanoparticles is tested in several media with different pH in order to underline their pH sensitive behavior. This is clearly described in Figure 4 referring to the release profile of diclofenac sodium used as a model to evaluate the response in neutral (PBS—pH 7.3, 1.0 M), slightly acid (distilled water—pH 6.3) and highly acid medium (HCl—pH 3, 0.001 M), respectively. In the first two cases, any significant drug amount is released due to the limited dissolution of chitosan carrier under the imposed pH conditions. Moving down to pH = 3, a two-step release mechanism may be recognized, which is characterized by an initial burst line followed by a slow sustained release.

## 3. Discussion

Micro and nanogels currently offer an interesting solution to design cell and macromolecular carriers for regenerative medicine and passive/active molecular targeting. In this context, chemical synthesis—*i.e*., monomer polymerization in solution—or physical assembly based on electrostatic interactions among polymer chains (*i.e*., coacervation) [18] or ionic gelation [19] have been often dropped for the use of organic solvents of chemical agents, being potentially hazardous to the environment as well as to physiological systems. More recently, novel technologies based on supercritical fluids have been also considered for their eco-sustainability and suitability for mass production, although several shortfalls mainly associated to production methods, high cost and increasing complexity of equipment [20]. Thanks to recent discoveries in nanotechnologies, it is possible to finely manipulate particle size and surface properties at micro and sub-micrometric scale for different applicative demands. At micrometric size, they can be optimized for a controlled and sustained drug release at the target site, improving the therapeutic efficacy and reducing side effects [21]. At the sub-micrometric scale, they can be used to overcome physiological barriers, such as biological membranes, being able to provide a more efficient extravasation through the vasculature, prolonged vascular circulation time, improved cellular uptake and endosomal escape [22].

Hence, electrospraying represents an innovative and cost-effective technique to directly incorporate cells or bioactive species into a polymeric carrier in a single step, in contrast to traditional methods requiring two or more steps to produce the final drug-loaded particles [23]. Different spraying modes (e.g., dripping, microdripping, simple-jet, single cone spraying and multiple cone spraying) have been recently investigated to design micro and nanoparticles for different use. They allow manipulating polymer solutions by the competition of the electric forces and the surface tension at the liquid/air interface and by the kinetic energy of the liquid leaving the nozzle. In all cases, polymer jet breaks up into fine droplets near the tip of nozzle as a function of the process conditions, due to varicose and/or whipping instability occurring after the Taylor cone formation. Once droplets are emitted from the tip, different scenarios may occur: Rayleigh disintegration or coulomb fission, once a polymer droplet is formed, solvent evaporation is predominant, polymer and charge concentrations drastically increase up to solidify the droplet, with the formation of micro or nanoparticles, which may aggregate themselves if solvent is not completely removed [24].

Starting from these studies, we have optimized ES process parameters including applied voltage, needle size, chitosan/acetic acid relative ratios and the collecting distance to properly control all the main microscopic phenomena, which address the formation of chitosan gels at different size scale, in order to design innovative micro or sub-microgels to carry out cells and/or macromolecules in biological microenvironment. Chitosan is a polycation whose primary amino groups can be protonated at low pH (pKa 6.5). It exhibits remarkable antibacterial, mucoadhesive, analgesic, hemostatic, biocompatible, and biodegradable properties [25]. Pancholi *et al*. have demonstrated that viscosity and surface tension of chitosan solution may influence particle diameter during electrospraying from few microns down to 500 nm [26]. Therefore, surface tension and electric conductivity of solvents play a critical role on the formation of polymer droplets. In the case of high surface tension, polymers cannot be atomized in air by electric forces but organic solvents are often required for the fabrication via ES due to their low surface tension [27]. In our case, the proper selection of solvents to dissolve chitosan represents a critical step to obtain micro/nanogels by ES, since the morphology of generated particles is highly dependent on the physicochemical properties of the solvent. In general, ES of polymer dissolved in solvents with low vapor pressure and high boiling temperature (e.g., N, N-dimethylformamide (DMF)) results in particles with smaller size and smoother surface morphology, characterized by a bimodal size distribution due to weaker polymer chain entanglement. In contrast, solvents with high vapor pressure, low boiling temperature, and, consequently, a faster evaporation rate (e.g., dichloromethane, acetic acid) may result in the formation of textured and/or highly porous surfaces, and even hollow structures. In fact, the fast solvent evaporation rate reduces the time that polymer chains require to re-arrange within the droplet during rapid solidification [28]. In our studies, chitosan nanoparticles show a uniform distribution of particles with sub-micrometric diameters by the fast removal of acetic acid solutions. However, in order to control shape and size distribution, water has been used as co-solvent system to provide a more stable formation of droplets, by controlling evaporation mechanisms and improving the interface with bioactive molecules. Indeed, solvent properties are crucial to optimize the fabrication via ES process of drug loaded particles. Indeed, they may interfere with the effective formation of entanglements occurring among polymer chains under the applied electric field, thus concurring to the final size and shape of particles as well as to the efficient encapsulation of molecular species with relevant outcomes for their use in pharmaceutical treatments. Moreover, they may also influence the peculiar behavior of chitosan to be sensitive to microenvironmental conditions. As reported in Figure 4, chitosan is readily soluble in dilute acidic solutions below pH 6.0, due to the presence of primary amino groups able to protonate at lower pHs, thus forming a water soluble cationic polyelectrolyte. Contrariwise, as the pH increases above 6, chitosan amines tend to deprotonate and the polymer loses its charge, thus becoming insoluble. Hence, the capability of solvents to mediate polymer chain interactions may contribute to influence the on-demand release mechanisms in acidic environmenta. Moreover, their capability to selectively respond to environmental change *in vitro* or *in vivo* is mainly related to the large quantities of amino groups on its chains [29] which are able to induce volume phase transitions from swollen to collapsed states or vise versa, with relevant effects on molecular release. Indeed, this peculiar feature is extremely important from applicative point of view, taking into account how drug release capacity of the particles significantly changes from a swollen to a collapsed state as a function of pH, thus rendering chitosan microgels and nanoparticles, particularly promising as carriers in acid microenvironment for oral delivery [30], tissue regeneration [31] and cancer therapy [32].

Conclusively, a sage evaluation of polymer/solvent coupling may be relevant to address all the typical mechanisms which regulate the intrinsic interaction among polymer chains mediated by electrical forces. It is well-known that ES of water or aqueous solutions may be limited by the coronal discharge (e.g., electrical break down) in the air. In order to improve local polar group interactions under the electric field forces, alternative strategies may be used: controlled inert gas (*i.e*., CO_2_) flowing at the needle tip may prevent the corona discharge [33]. However, conductivity or dielectric constant of liquid plays the main role by affecting the cone-jet mode [27]. As a consequence, pure water cannot be commonly used to atomize particles at sub-micrometric size scale due to the occurrence of other phenomenon negatively influencing electric forces. Certainly, water surface tension is so high that the electric field strength needed to form a jet higher than air breakdown strength and—at the cone apex—a corona discharge may be observed. In this case, surfactant agents cannot be successfully used because of much longer diffusion times at the surface, due to its lower surface tension and jet formation times comparable with electrical relaxation time [34]. However, the dipole formation of highly polar water molecules is really interesting for the atomization of chitosan microgels by EHDA process. In this case, charges are transferred/immobilized to the surface of cone and jet, thus causing jet break-up, and high flow rates conditions avoid any overcharge of the polymer droplet, promoted by the presence of easily polarized water molecules, thus inducing the polymer flow breaking in balloons of few hundred microns in size. Case by case, the addition of water soluble solvents (*i.e*., ethanol, isopropyl alcohol, acetone) may accelerate evaporation mechanisms, thus concurring to the final size of polymer droplets breaking prior to the fission. Meanwhile, the control of chitosan concentration or the addition of other polymers (*i.e*., polyethyleneglycol, polyvinylalcohol) at low concentration, may increase the solution viscosity, which is crucial to control fluidodynamic instabilities (*i.e*., varicose effect) associated with droplet formation.

## 4. Conclusions and Future Trends

ES technologies offer a facile and robust method to produce micro or nanogels with well-controlled size, morphology, structure, and shapes for various uses as carriers in cell and drug therapy. By properly set materials properties and process conditions, they allow generating—by a single step process—monodisperse gels with differently-sized scales. Recent studies have just demonstrated the possibility to fabricate various multi-layered structured gels by tailored process setup configurations based on the use of simply coaxial [35] or triple coaxial systems [36]. The use of modified co-axial ES systems could be optimized also to fabricate biphasic Janus gels or nanocolloids with nanoscale anisotropy by side-by-side technologies [37], moving towards multicompartmental systems including pie-shaped, asymmetric, striped, and rosette compartment configurations. Therefore, ES technologies could be extremely interesting not only for the fabrication of smart drug delivery systems, but also to design new micro/sub-micrometric devices able to successfully interface and interact with human cells for new biomedical applications (*i.e*., therapeutics, medicals, analytics, diagnostics). In this perspective, new intriguing strategies should be continuously explored to design “effective” living systems, which integrate actives, genes and cells into micro-atomized or electrosprayed particles, with the aim of design highly complex 3D models for the repair or replacement of damaged or simply aged tissue portions.

## 5. Materials and Methods

### 5.1. Microgels

Low molecular weight CHI (75%–85% deacetylated, Aldrich) is dissolved in an aqueous solution of acetic acid (C_2_H_4_O_2_, Aldrich) at different concentrations via magnetic stirring for 72 h. Aqueous chitosan solutions are processed by NF500 (MECC, Japan), applying high voltage on the polymer jet dispensed through a 27G needle tip. The polymer solution (2–3 *wt*/*v* %) is loaded into a syringe, fitted with a conductive steel capillary and infused at several flow rates by a syringe pump. A voltage from 18 to 30 kV is applied and the electrosprayed microspheres are collected directly into a sodium hydroxide (NaOH) solution at a given distance from the tip. The effects of concentration, flow rate and voltage, on drop formation and consequently on the shape and size of resulting microparticles, are qualitatively investigated by optical microscopy (Olympus BX51) and quantitatively by using image analysis software (Image J, v.1.37; NIH, Bethesda, MD, USA).

### 5.2. Nanoparticles

Electrosprayed CHI nanoparticles are obtained by dissolving CHI (75%–85% deacetylated, Aldrich) in an aqueous solutions of 70%, 80%, 90% acetic acid (C_2_H_4_O_2_, Aldrich) at different concentrations (from 1 to 3 *wt*/*v* %) via magnetic stirring for 48 h at room temperature. CHI solutions are processed via electrospraying (NANON01, MECC, JAPAN) by properly setting process parameters to obtain sub-micrometric round-like particles. The polymer solution is placed in a 5 mL syringe and is continuously pushed by the syringe pump at several flow rates (0.1–0.3 mL/h) using a steel nozzle (18–27 Ga) which is connected to the high-voltage power supply to generate a potential difference (13–28 kV) between nozzle and ground collector. Lastly, nozzle/collector distance is fixed between 7 to 10 cm to prevent clogging phenomena at the needle tip due to fast solvent evaporation.

The morphology of electrosprayed particles is characterized by a field emission scanning electron microscope (FESEM, QuantaFEI200, The Netherlands) and the size distribution of polymer particles were measured using image analysis software (Image J v.1.37).

Moreover, a model molecule (*i.e*., diclofenac sodium salt, Sigma Aldrich, Italy) is loaded into chitosan particles to investigate the drug release profile in different media at several pH—from neutral to acidic values. Lastly, release profiles are measured by UV spectrophotometry at λ_max_ of 380 nm.
